# *Lactobacillus lactis* CKDB001 ameliorate progression of nonalcoholic fatty liver disease through of gut microbiome: addendum

**DOI:** 10.1080/19490976.2020.1829449

**Published:** 2020-11-01

**Authors:** Na Young Lee, Hyun Chae Joung, Byoung Kook Kim, Byung Yong Kim, Tae Sik Park, Ki Tae Suk

**Affiliations:** a Institue for Liver and Digestive Disease, Hallym University, Chuncheon-si, Republic of Korea; b Chong Kun Dang Bio Research Institute, CKDBiO, Ansan-si, Republic of Korea; c ChunLab, Inc., Microbiome Department, Seoul, Republic of Korea; d Department of Life Science, Gachon University, Sungnam, Republic of Korea; e Department of Internal Medicine, Hallym University College of Medicine, Chuncheon, Republic of Korea

**Keywords:** Word, *Lactobacillus delbrueckii* subsp. *lactis*, nonalcoholic fatty liver disease, 16s rRNA sequencing, Whole gene sequencing

## Abstract

According to our recent study (N.Y. LEE et al. Gut Microbes 2020; 11:882–99.)^1^, we reported that *Lactobacillus* and *Pediococcus* ameliorate progression of nonalcoholic fatty liver disease through modulation of the gut microbiome. According on the analysis method (Previous: 16s rRNA sequencing and Recent: whole gene sequencing), the probiotics named *Lactobacillus bulgaricus* that we used in the experiment was identified as *Lactobacillus delbrueckii* subsp. *bulgaricus* through 16s rRNA sequencing analysis. Recently, we performed a clearer analysis with whole gene sequencing to proceed with the clinical trial, it was identified as *Lactobacillus delbrueckii* subsp. *lactis* by whole gene sequencing. Therefore, we inform that the subspecies have been changed to *lactis* through WGS. Read *L. bulgaricus* in the previous paper as *L. lactis*. In this addendum, the results of the change to *L. lactis* are summarized, and descriptions have been added to Materials & methods and Discussion.

## Addendum for

*Lactobacillus* and *Pediococcus* ameliorate progression of nonalcoholic fatty liver disease through modulation of the gut microbiome.^1^ DOI: 10.1080/19490976.2020.1712984

## Results

### Liver/body weight ratio

**Figure 1.**
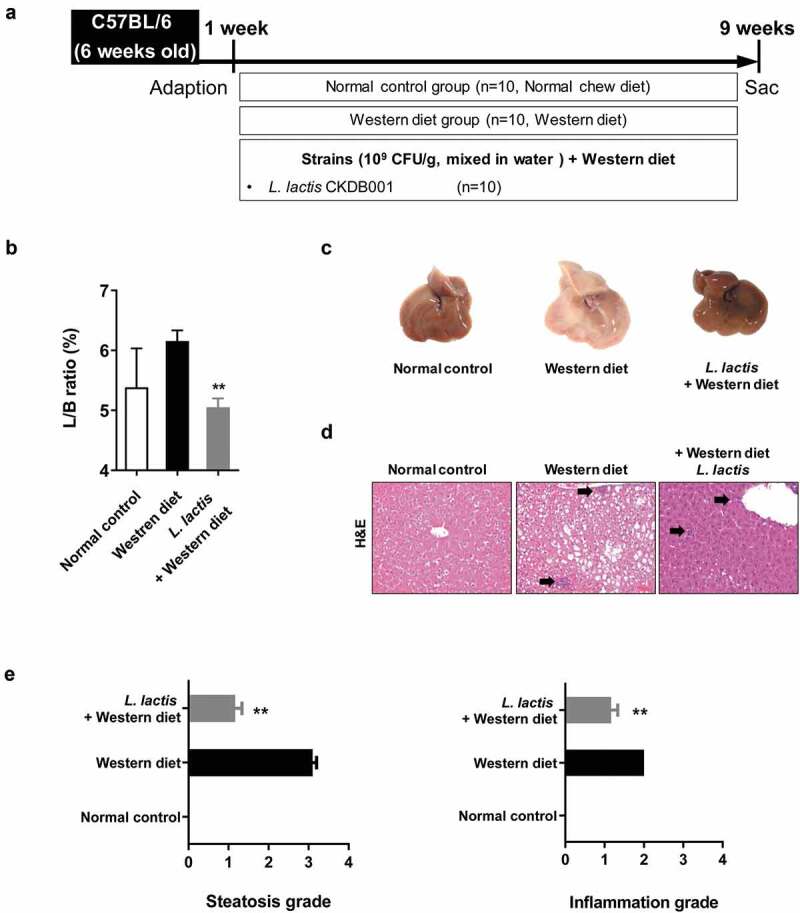
Animal experiment results. (a) Flow chart of the animal experiment. (b) Liver/body weight ratio in mice. (c) Gross specimen of mice liver. (d) Pathological effects of *L. lactis* on the liver (Hematoxylin and Eosin stain). ×200 (e) Steatosis grade and (f) Inflammation grade. *p < 0.05, **p < 0.001

In the analysis of the liver/body weight ratio (%), the *L. lactis* group (5.05 ± 0.46) was significant improvement compared with the Western diet group (6.16 ± 0.56) (*p* < 0.001) (Figure 1b).

### Liver function test and serum cholesterol level

The Western diet group demonstrated significantly elevated liver enzyme levels compared with the normal control group (*p* < 0.05). The mean levels of AST in the *L. lactis* group (106 ± 22 U/L) was lower than the Western diet group (181 ± 42 U/L) (*p* < 0.05). Also, *L. lactis* group (179 ± 45) decrease in almost half the total cholesterol level in serum compared with the Western diet group (353 ± 46) (*p* < 0.05) (Table 2).

**Table 1.** Information of *L. lactis* CKDB001

Strains	Number of bacteria (CFU/g)	Characteristics	Known roles in disease
*L. lactis* CKDB001	1.40*10^11^	Gram-positive	Decrease fasting blood glucose^1^ Prevent the adhesion and invasion of pathogenic bacteria^3^

CFU, colony forming unit.

**Table 2.** Liver function test and serum cholesterol level of mice

Variables (mean ± SD)	Normal control	Western diet	*L. lactis+ Western diet*
AST (U/L)	95 ± 49	181 ± 42	106 ± 22*
ALT (U/L)	59 ± 20	77 ± 20	55 ± 12*
Cholesterol (mg/dL)	83 ± 26	353 ± 46	179 ± 45*

*p < 0.05, compared with the Western diet control.

SD, standard deviation; n, number; AST, aspartate aminotransferase; ALT, alanine aminotransferase.

**Table 3.** Universal primers for V3 – V4 region of the bacterial 16S rRNA gene

Sample information	Forward primer (341 F) 5’-> 3’	Reverse primer (805 R) 5’-> 3’	Forward index	Reverse index
Normal control 1	CCTACGGGNGGCWGCAG	GACTACHVGGGTATCTAATCC	CTCTCTAT	TCGCCTTA
Normal control 2	CCTACGGGNGGCWGCAG	GACTACHVGGGTATCTAATCC	CTCTCTAT	CTAGTACG
Normal control 3	CCTACGGGNGGCWGCAG	GACTACHVGGGTATCTAATCC	CTCTCTAT	TTCTGCCT
Western diet 1	CCTACGGGNGGCWGCAG	GACTACHVGGGTATCTAATCC	CTCTCTAT	GCTCAGGA
Western diet 2	CCTACGGGNGGCWGCAG	GACTACHVGGGTATCTAATCC	CTCTCTAT	AGGAGTCC
Western diet 3	CCTACGGGNGGCWGCAG	GACTACHVGGGTATCTAATCC	CTCTCTAT	CATGCCTA
*L. lactis* 1	CCTACGGGNGGCWGCAG	GACTACHVGGGTATCTAATCC	CTCTCTAT	TCCTCTAC
*L. lactis* 2	CCTACGGGNGGCWGCAG	GACTACHVGGGTATCTAATCC	CTCTCTAT	TCATGAGC
*L. lactis* 3	CCTACGGGNGGCWGCAG	GACTACHVGGGTATCTAATCC	CTCTCTAT	CCTGAGAT

### Pathologic findings

**Figure 2.**
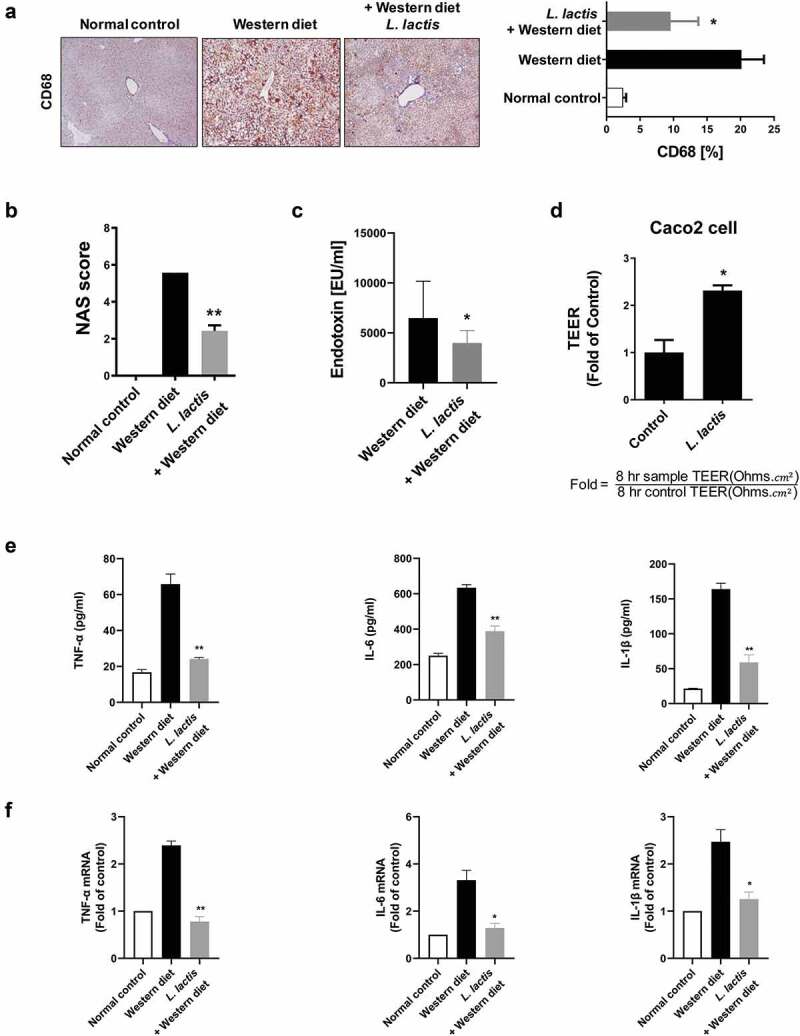
Gut-Liver axis analysis and pro-inflammatory cytokines. (a) Immunohistochemical analyses for CD68 in representative cases. ×400. (b) NAFLD Activity Score (NAS). (c) Stool endotoxin level. (d) Trans-epithelial electrical resistance (TEER) measurement. (e) Enzyme-linked immunosorbent assay. (f) Real-time reverse transcription-polymerase chain reaction. *p < 0.05, **p < 0.001

Moderate to marked macrovesicular steatosis developed in the Western diet group (Figure 1d). Additionally, large fat vacuoles displaced the nuclei to the edges of the cells, representing minimal to mild mixed macro/microvesicular steatosis. The grade in *L. lactis* group was lower than the Western diet group (*p* < 0.001). Regarding to the hepatitis, the Western group had an inflammation grade of 2. However, the *L. lactis* group showed significance reduced the inflammation grade that minimal to mild inflammation was demonstrated (*p* < 0.001) (Figure 1e). Concerning the NAFLD activity score (NAS), the *L. lactis* group (2.4 ± 0.8) significantly decreased compared with the Western diet group (5.0 ± 1.6) (*p* < 0.001) (Figure 2b).

Immunohistochemical analysis for CD68, marker for macrophages, in representative cases. Mean value of positive stained area measured in 10 random area of liver. The normal control (2.4 ± 1.6%), the Western diet (20.1 ± 6.1%) and the *L. lactis* (9.6 ± 6.6%). The *L. lactis* group showed a significant reduction of the stained area compared to the area of the Western diet group (*p* < 0.05) (Figure 2a).

### Stool endotoxin levels

Elevated levels of endotoxin levels in the Western diet group (6,475 ± 5,231 EU/ml) were significantly reduced in the *L. lactis* group (3,979 ± 1,779 EU/ml), (353 ± 46) (*p* < 0.05) (Figure 2c).

### Trans-epithelial electrical resistance measurement

Caco-2 cells were cultured for complete confluence and coincubated with bacterial suspension in MEM media for 8 hours. Treatment of Caco-2 cells with the *L. lactis* CKDB001 increased TEER values by 2.3-fold, respectively, when compared with those of no treatment controls (*p* < 0.05) (Figure 2d). This result suggests that the *L. lactis* CKDB001 increased gut barrier function between Caco-2 cells and might regulate the endotoxin infiltration in the gut.

### Inflammatory cytokines in liver

Enzyme linked immunosorbent assays revealed elevated levels of TNF-α, IL-1β, and IL-6 in the Western diet group (65.8 ± 7.9, 163.8 ± 12.2, and 633.6 ± 25.8 pg/ml, respectively), and these levels were significantly reduced in the *L. lactis* (24.2 ± 1.01, 58.9 ± 15.3, and 388.3 ± 42.0 pg/ml, respectively) (*p* < 0.001) (Figure 2e).

Real-time reverse transcription-polymerase chain reaction showed the same pattern results. The *L. lactis* group was protected against the Western diet group that showed improvement in the levels of inflammatory TNF-α, IL-1β, and IL-6 (*p* <0 .05) (figure 2f).

### Stool analysis for metagenomics

**Figure 3.**
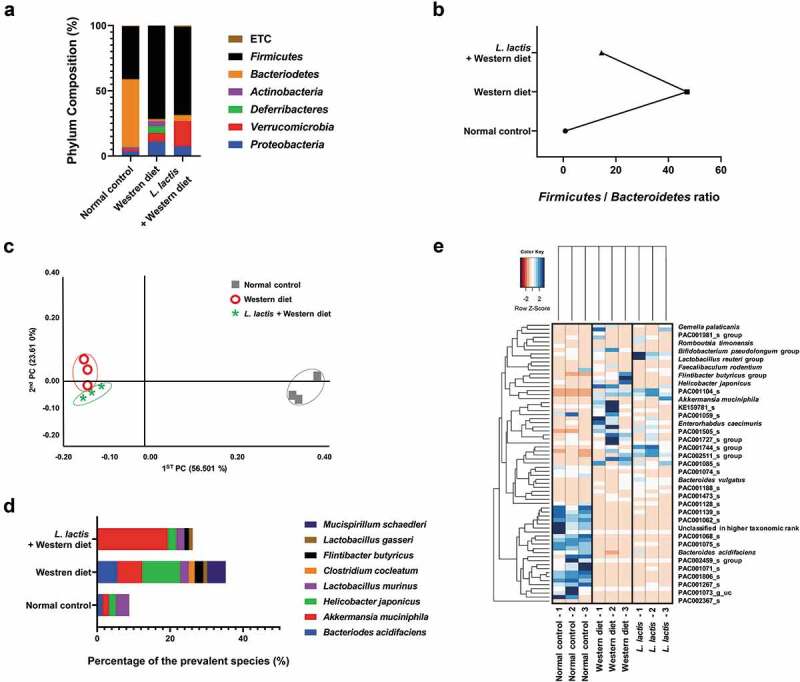
Animal stool analysis. (a) Phylum composition analysis in each group. (b) Firmicutes/Bacteroidetes ratio. (c) Analytics for beta diversity for the relationship between microbiome taxonomic profiling (principle coordinates analysis, Jensen-Shannon, species, include unclassified OTUs). (d) Comparison of prevalent species. (e) Heatmap for composition of species diversity in mice stool

**Figure 4.**
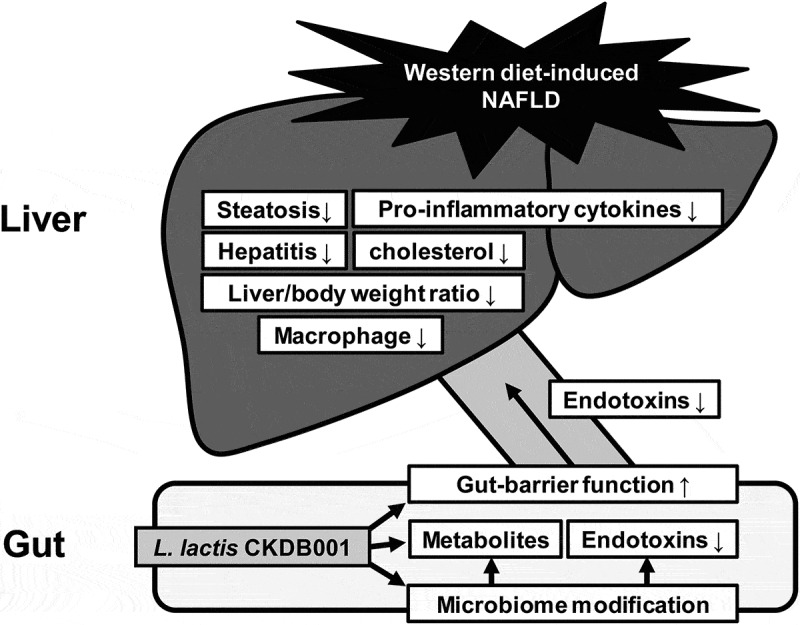
Schematic overview of gut-liver-axis modulation by *L. lactis.* In the western diet-induced NAFLD model, the gut microbiome is modulated due to the intake of*L.lactis* CKDB001, which is regulated in the progression of fatty liver

In the phylum analysis of animal stool samples, the compositions of *Proteobacteria, Verrucomicrobia, Deferribacters, Actinobacteria, Bacteroidetes, Firmicutes*, and others (Under 1% on average) were different that the normal control [3%, 2%, 0%, 2%, 52%, 41%, and 1%], the Western diet [1%, 7%, 5%, 4%, 2%, 72%, and 0%], and the *L. lactis* [7%, 19%, 0%, 0%, 5%, 68%, and 1%] (Figure 3a). *Firmicutes* and *Bacteroidetes* are major phylum of domain bacteria and are dominant in human gut microbiota. The *Firmicutes*-to-*Bacteroidetes* ratio (F/B ratio) is correlated with obesity and other diseases. Elevated F/B ratio in the western diet group (47.1) was decreased in *L. lactis* group (Figure 3b). We evaluated the difference between groups by utilizing distance measures of beta diversity. In the analytics for beta diversity for the relationship between microbiome taxonomic profiling, each group showed a different location (Figure 3c). In the comparison of prevalent species that commonly exist in groups, the composition was different among groups (Figure 3d). The species with high percentage in each group (but not all species) were selected and compared by heatmap (Figure 3e). Interestingly, *A. muciniphila* which is known to be beneficial microbiome, was increased in the *L. lactis* group [19.4%] compared with the Western diet group (Figure 4, Table 3).

## Discussion

In recent study, intake of *Lactobacillus delbrueckii* subsp. *lactis* PTCC1057 showed effective results in STZ-induced diabetes mouse model.^2^ Oral administration of *L. lactis* PTCC1057 has been shown to significantly reduce serum glucose compared to the diabetes control group. And it led to decrease in fasting blood glucose and fetuin-A levels and increase in serum sestrin 3 levels. This study suggests *L. lactis* PTCC1057 as a candidate probiotic for diabetes. In our study, *L. lactis* group have improvements in liver/body weight ratio compared with the Western diet group. Moreover, it decreased steatosis grade, inflammation grade, and NAS in liver. Altogether, these data showed that *L. lactis* are effective for NAFLD.

Also, other studies say that intestinal cell infection by B. cereus induces the production of pro inflammatory cytokines by dendritic cells (IL-8, IL-6, TNF-α), then *L. lactis* CIDCA 133 effectively regulates cellular responses in both infected epithelial and dendritic cells.^3^ In our results, intake of *L. lactis* CKDB001 effectively decreased TNF-α, IL-6, and IL-1β level in the liver tissue. Moreover, *L. lactis* R4 *prevents Salmonella typhimurium* SL1344-induced damage to tight junctions and adherens junctions.^4^ The results of this study showed that *L. lactis* R4 increased the expression of ZO-1 and occludin to maintain cell junctions and mucosal barriers and prevent the adhesion and invasion of pathogenic bacteria.

In our study, intake of *L. lactis* CKDB001 modulates gut microbiome to protects the liver, improves liver function, and reduces inflammatory cytokines. Although study on the mechanism for this is still lacking, these outstanding effects suggest that by *L. lactis* CKDB001 as a promising therapeutic candidate for NAFLD.

The 16S rRNA microbe is about 1,500bp and the base sequence is different for each species. There are 9 variable regions with high variability (V1-V9), and there are constant regions with the same nucleotide sequence in between. 16S rRNA sequencing is a method to check the base sequence of the variable region through PCR amplification by making a primer according to the constant region.^5^ It is widely used because it can easily identify strains up to the species level in a short time, but it is difficult to distinguish subspecies due to the short nucleotide sequence that can be used for analysis.^6^ Most microbes have multiple copies of the gene in a single genome, which obviously interferes with the final analysis of the sequence data.^7^ In this reason, a result of analyzing the requested sample, the *Lactobacillus delbreuckii* subspecies *bulgaricus* strain ATCC 11842 16S ribosomal RNA gene was analyzed with the highest similarity (99%) in 2015. It also showed homology with *L. sunkii* and *L. indicus* by NCBI Gene Bank Data.

WGS is a method of analyzing nucleotide sequences and genes through microbial DNA extraction, DNA fragmentation, DNA library production, DNA sequence analysis de novo assembly, and gene annotation. WGS also has the advantage of being able to retrieve 16 rDNAs from the genome and use them for species profiling and adding to database. Through WGS analysis, all nucleotide sequences can be analyzed, and strain identification is also possible precisely to subspecies. WGS tends to show more accurate results compared to 16s rRNA sequencing. It is conducted via shotgun sequencing, and any limitations associated with that technology also apply to WGS. In the case of the previous strain, which appeared as *L. bulgaricus*, it was identified as *L. lactis* and 2,052,085bp sequence analysis was completed in 2020.

The 16S rRNA database registered with NCBI has been registered since the beginning of the genomic analysis, and the quantity is vast but contains inaccurate results. Recently as the NGS analysis method has been further developed, accurate strain identification results are constantly being updated, so the databases in 2015 and 2020 show differences inaccuracy. Therefore, since WGS in 2020 is a more accurate analysis method than 16S rRNA sequencing in 2015, so we modified *L. bulgaricus* to *L. lactis* to clarify this.

## Materials and methods

### Strain

*L. lactis* are lactic and bacteria that have been isolated from various including sour milk, cheese, healthy Korean adults (30-year-old), and newborn baby feces, respectively. The strain incubated under anaerobic conditions at 37°C for 24 h. Stocks of each strain were prepared by mixing the culture broth with an equivalent 20% skim milk solution and then storing at −80°C until use The baseline characteristics of the L. lactis are reported in Table 1.

### 16s rRNA sequencing

Genomic DNA of probiotics was isolated using a genomic DNA isolation kit (GeneALL, Seoul, Republic of Korea), following the manufacturer’s protocol. PCR amplification of the 16S rRNA gene from probiotics was performed with the primers 27 F and 1492 R and sequencing of the PCR product was done with 27 F and 785 F (5′-GGATTAGATACCCTGGTA-3′) primers to get a partial sequence of the 16S rRNA gene. Sequencing service was provided by Solgent Co. Ltd. (Seoul, Republic of Korea). The sequence was searched for similarities in the rRNA/ITS databases using the BLAST program.

### Whole gene sequencing (WGS)

DNA was extracted using a MG genomic DNA purification kit (MGmed, Seoul, Republic of Korea) according to the manufacturer’s instructions. A sample of High-quality and high-molecular-weight DNA is required to prepare size-selected approximately 20 kb SMRTbell templates. We used NanoDrop spectrophotometer (Thermo Scientific, Waltham, MA, USA) and Quant-IT PicoGreen (Invitrogen, Carlsbad, CA, USA) to measure the concentration of genomic DNA. Evaluation of gDNA quality using Bio-Rad electrophoresis system.

For PacBio RSII sequencing, 8 ug of input genomic DNA was used for 20 kb library preparation. For gDNA where the size range was less than 17kb, we used the Bioanalyzer 2100 (Agilent, Santa Clara, CA, USA) to determine the actual size distribution.

The library insert sizes were in the optimal size range, we sheared gDNA was sheared with g-TUBE (Covaris Inc., Woburn, MA, USA) and purified using AMPurePB magnetic beads (Beckman Coulter Inc., Brea, CA, USA) if the apparent size was greater than 40 kb.

Total 10uL library was prepared using PacBio DNA Template Prep Kit 1.0. SMRTbell templates were annealed using PacBio DNA/Polymerase Binding Kit P6. The PacBio DNA Sequencing Kit 4.0 and 8 SMRT cells were used for sequencing. SMRT cells (Pacific Biosciences, Menlo Park, CA, USA) using C4 chemistry and 240 min movies were captured for each SMRT cell using the PacBio RS II (Pacific Biosciences) sequencing platform. The subsequent steps are based on the PacBio Sample Net-Shared Protocol, which is available at http://pacificbiosciences.com/.

### Genome assembly

Contigs were assembled using CANU v1.7 with long reads.

### Genome annotation

For annotation of prokaryotic genome, we annotated genome using Prokka v1.1.0. the gene models were predicted by ORF finding method using prodigal v2.6.2. And then tRNA, rRNA, repetitive sequence was identified with Aragon v1.2.36 (tRNA), barrnap v0.6 (rRNA), and minced v0.2.0 (repetitive sequence). For functional annotation, genes were searched against the UniProt and NCBI RefSeq databases using BLASTP v2.2.29+ with an E-cut off value of 1E-6. Protein domains were also searched against the Pfam using HMMER 3.1b1.

### Taxonomic identification

The sequence was identified using NCBI Whole-genome shotgun contigs (WGS) database.

### Other materials and methods

For other experimental materials and methods are the same as the author’s previous paper (N.Y. LEE et al. Gut Microbes 2020; 11:882–99.).^1^
